# Efficient conversion of xylose to ethanol by stress-tolerant *Kluyveromyces marxianus* BUNL-21

**DOI:** 10.1186/s40064-016-1881-6

**Published:** 2016-02-27

**Authors:** Sukanya Nitiyon, Chansom Keo-oudone, Masayuki Murata, Noppon Lertwattanasakul, Savitree Limtong, Tomoyuki Kosaka, Mamoru Yamada

**Affiliations:** Applied Molecular Bioscience, Graduate School of Medicine, Yamaguchi University, Ube, 755-8505 Japan; Department of Biology, Faculty of Science, National University of Laos, Vientiane, Lao PDR; Department of Microbiology, Faculty of Science, Kasetsart University, Bangkok, 10900 Thailand; Department of Biological Chemistry, Faculty of Agriculture, Yamaguchi University, Yamaguchi, 753-8515 Japan

**Keywords:** *Kluyveromyces marxianus*, Thermotolerant yeast, Ethanol fermentation on xylose, Stress resistance

## Abstract

The fermentation ability of thermotolerant *Kluyveromyces marxianus* BUNL-21 isolated in Laos was investigated. Comparison with thermotolerant *K. marxianus* DMKU3-1042 as one of the most thermotolerant yeasts isolated previously revealed that the strain possesses stronger ability for conversion of xylose to ethanol, resistance to 2-deoxyglucose in the case of pentose, and tolerance to various stresses including high temperature and hydrogen peroxide. *K. marxianus* BUNL-21 was found to have ethanol fermentation activity from xylose that is slightly lower and much higher than that of *Scheffersomyces stipitis* (*Pichia stipitis*) at 30 °C and at higher temperatures, respectively. The lower ethanol production seems to be due to large accumulation of acetic acid. The possible mechanism of acetic acid accumulation is discussed. In addition, it was found that both *K. marxianus* strains produced ethanol in the presence of 10 mM hydroxymethylfurfural or furfural, at a level almost equivalent to that in their absence. Therefore, *K. marxianus* BUNL-21 is a highly competent yeast for high-temperature ethanol fermentation with lignocellulosic biomass.

**Electronic supplementary material:**

The online version of this article (doi:10.1186/s40064-016-1881-6) contains supplementary material, which is available to authorized users.

## Background

Compared with *Saccharomyces cerevisiae,* which is widely used in ethanol fermentation industries, *Kluyveromyces marxianus* has advantageous potentials for application in ethanol production. First, *K. marxianus* is thermotolerant and is able to efficiently produce ethanol at high temperatures (Limtong et al. 2007), allowing us to develop high-temperature fermentation technology that will reduce cooling cost, enable efficient simultaneous saccharification and fermentation, reduce the risk of contamination and enable stable fermentation even in tropical countries (Anderson et al. 1986; Banat et al. 1998; Limtong et al. 2007). Second, the yeast can assimilate various sugars including xylose, arabinose, sucrose, raffinose and inulin in addition to several hexoses (Lertwattanasakul et al. 2011; Rodrussamee et al. 2011). This broad spectrum in sugar assimilation capability is very beneficial for conversion of biomass consisting of various sugars to ethanol. Third, the yeast exhibits a relatively weak glucose repression on utilization of some sugars including sucrose (Lertwattanasakul et al. 2011) and is thus highly suitable for biomass such as sugar cane juice including glucose, fructose and sucrose as main sugars.

For application of such beneficial characteristics, however, some crucial points regarding *K. marxianus* should be improved. One point is a relatively strong glucose repression on utilization of other sugars including xylose (Rodrussamee et al. 2011). Another point is its low conversion ability of xylose to ethanol. The ethanol fermentation potential and sugar utilization profile of thermotolerant *K. marxianus* DMKU3-1042 have been extensively studied (Limtong et al. 2007; Lertwattanasakul et al. 2011; Rodrussamee et al. 2011; Pimpakan et al. 2012). The strain is capable of producing ethanol from all sugar components in lignocelluloses except arabinose. Lignocellulose, which is a second-generation biomass for biofuels, contains glucose, xylose and arabinose as abundant sugars. Tremendous exploration (Toivola et al. 1984; Barnett et al. 2000; Kurtzman et al. 2011) has revealed that only a few yeast species can efficiently ferment D-xylose to ethanol. Xylose fermentation ability of *K. marxianus* strains isolated previously, however, is much lower than those of *Scheffersomycesstipitis* (*Pichiastipitis*) and *Spathaspora passalidarum* (Nguyen et al. 2006; Krahulec et al. 2012; Su et al. 2015), and the yield of ethanol production varies in different strains (Margaritis and Bajpai 1982; Banat et al. 1996; Wilkins et al. 2008; Rodrussamee et al. 2011). Gene engineering has thus been attempted to improve the ethanol productivity from xylose in *K*. *marxianus* (Wang et al. 2013; Zhang et al. 2013). In addition, it is known that toxic compounds, including hydroxymethylfurfural (HMF) and furfural, that are generated in the process of hydrolysis of lignocellulosic materials prevent the growth or fermentation efficiency of microbes (Mussatto and Roberto 2004; Behera et al. 2014), and strains that are resistant to them should thus be developed. It is notable that some ethanol-fermenting microbes such as *S. stipitis* are resistant to furfural at low concentrations (Nigam 2001).

In this study, we characterized a newly isolated strain of *K. marxianus,* BUNL-21 strain, which is one of the most thermotolerant yeasts screened in Laos. The BUNL-21 strain was shown to be superior to the DMKU3-1042 strain, which is one of the most thermotolerant and efficient *K. marxianus* strains isolated previously, in terms of the conversion activity of xylose to ethanol, resistance to 2-deoxyglucose in the case of pentose and tolerance to various stresses. In addition, we found efficient conversion of xylose to ethanol in the presence of HMF or furfural in *K. marxianus* strains tested. We also noticed a large accumulation of acetic acid on the xylose medium, which was more than that of ethanol, and we discuss its accumulation in comparison with *S. stipitis.*

## Methods

### Yeast strains

The yeast strains used in this study were thermotolerant *K. marxianus* BUNL-21 (NITE P-01739) and DMKU3-1042 (NITE AP-283), which were isolated in Lao PDR (Results for its screening and identification will be published elsewhere.) and Thailand (Limtong et al. 2007), respectively, and *S. stipitis* CBS 5773. They were stored in YPD medium (10 g/l yeast extract, 20 g/l peptone and 20 g/l glucose) supplemented with 10 % (v/v) glycerol at −80 °C.

### Analysis of ethanol fermentation ability

To investigate the ethanol fermentation ability of *K. marxianus* strains, YP medium (10 g/l yeast extract and 20 g/l peptone) supplemented with 20 g/l of glucose (Glc) or xylose (Xyl), designated as YPD or YPXyl, respectively, was used. When mixed sugars were used, YP media supplemented with 2 or 20 g/l Glc and 20 g/l of Xyl, designated as YPXyl + 0.2 % Glc and YPXyl + 2 % Glc, respectively, were used. Cells were precultured in 30 ml of YPXyl or YPD medium in a 100-ml Erlenmeyer flask sealed with aluminium foil at 30 °C under a rotary shaking condition at 160 rpm for 18 h. The preculture was inoculated to 30 ml fresh medium of YPXyl or YPD in a 100-ml Erlenmeyer flask sealed with aluminium foil at the initial OD_660_ value of 0.1 and incubated at 30 or 37 °C under a shaking condition at 160 rpm. The closure with aluminum foil may reduce oxygen transfer to flasks (Chain and Gualandi 1954) and cause an oxygen-limited condition. Yeast growth was determined by measuring optical density at 660 nm on a UVmini-1240 spectrophotometer (Shimadzu, Japan) and the values were converted to dry cell weight (DCW, g/l) by using conversion factors in equations in Additional file 1: Fig. S1. To determine sugar and ethanol concentrations in culture media, cultures were sampled and centrifuged at 14,000 rpm for 5 min. The supernatant was then subjected to quantitative analysis of sugars on a high-performance liquid chromatography apparatus (Hitachi, Japan) with a GLC610-S Gel pack column (Hitachi) connected to a refractive index detector Model L-2490 (Hitachi) in the mode of 0.5 ml/min eluent of deionized water at 60 °C. Acetic acid was analyzed on a high-performance liquid chromatography apparatus (Hitachi) with a GLC610-H Gel pack column (Hitachi) connected to a UV detector Model L-2400 (Hitachi) in the mode of 1 ml/min eluent of 0.1 % phosphoric acid at 60 °C. Ethanol concentration was analyzed by a gas chromatography GC-2014 apparatus (Shimadzu) with a glass column packed with PEG-20M (Shimadzu) connected to a flame ionization detector. We performed each experiment at least three times and obtained similar results.

### Analysis of stress resistance and glucose repression

Cells were grown at 30 °C for 18 h in YPD medium, harvested, washed with distilled water, and resuspended in distilled water (l × 10^7^ cells/ml). The cells were then tenfold sequentially diluted and spotted onto YPD agar plates supplemented with 5 mM hydrogen peroxide (H_2_O_2_), 8 % (w/v) ethanol, 35 % (w/v) Glc, 10 mM furfural or 10 mM HMF. The plates were incubated at 30 °C for 48 h. To examine glucose repression on the assimilation of other sugars, 2-deoxyglucose (2-DOG) as a glucose analog was used. Cells were spotted onto YP agar plates with or without 0.01 % 2-DOG in the presence of different carbon sources: 20 g/l mannose (YPMan), 20 g/l galactose (YPGal), 20 g/l xylose (YPXyl) and 20 g/l arabinose (YPAra). These plates were incubated at 30 or 37 °C for 48 h. We performed each experiment at least three times and obtained similar results.

## Results

### Comparison of ethanol fermentation ability on Xyl in *K. marxianus* BUNL-21 with those in K. marxianus DMKU3-1042 and *S. stipitis* at different temperatures

**Fig. 1:**
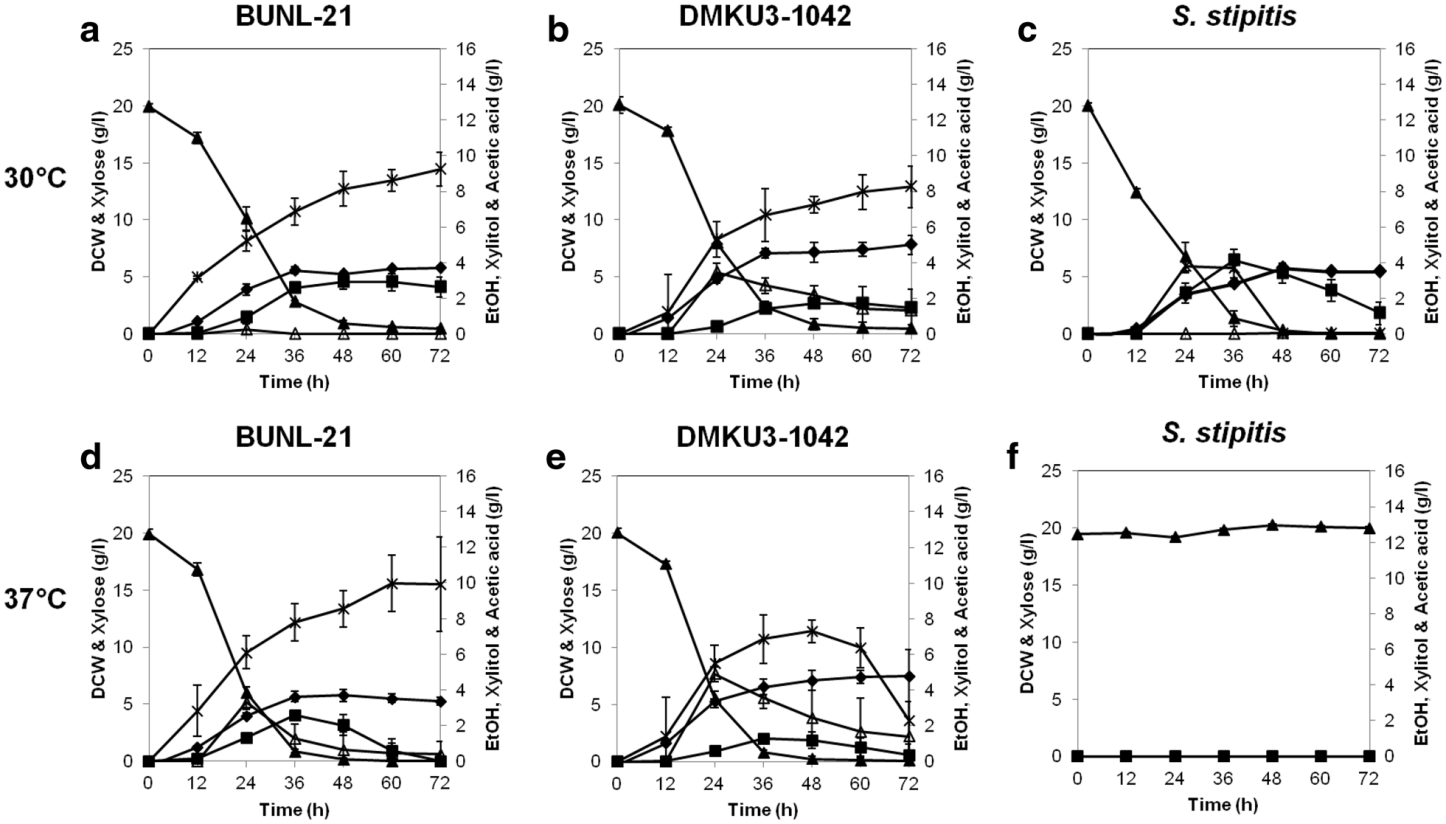
Growth and metabolite profiles of *K. marxianus* BUNL-21 and DMKU3-1042 and *S. stipitis* in YPXyl medium at 30 and 37 °C. *K. marxianus* BUNL-21 (**a**,** d**) and DMKU3-1042 (**b**,** e**) and *S. stipitis* (**c**, **f**) were grown in YPXyl medium at 30 °C (**a**–**c**) or 37 °C (**d**–**f**) under a shaking condition at 160 rpm, and samples were taken every 12 h until 72 h of incubation. The dry cell weight (*filled diamonds*) and concentrations of Xyl (filled triangles), ethanol (filled squares), xylitol (*open triangles*) and acetic acid (*crosses*) are shown.* Bars* represent the ±SD of values from experiments performed in triplicate

Comparison of several *K. marxianus* strains allowed us to select BUNL-21 as the highest ethanol fermenting strain on Xyl. Its growth and ethanol fermentation ability were thus compared with those of the DMKU3-1042 strain, which is one of the most efficient strains that has been analyzed in detail (Limtong et al. 2007; Rodrussamee et al. 2011). In the case of Xyl as a carbon source, its assimilation and ethanol fermentation of DMKU3-1042 have been shown to be greatly reduced at high temperatures (Rodrussamee et al. 2011). Ethanol fermentation of DMKU3-1042 under a temperature-uncontrolled condition was performed, and the maximum temperature for the fermentation process was found to be 35 °C (Murata et al. 2015). We thus compared the effects of temperatures (30 and 37 °C) on ethanol fermentation from Xyl in BUNL-21, DMKU3-1042 and *S. stipitis* (Fig. 1; Table 1). At 30 °C, biomass yields of BUNL-21 and *S. stipitis* were found to be lower than that of DMKU3-1042 in YPXyl medium. *S. stipitis* completely consumed Xyl within 48 h, but a trace amount of Xyl remained in both *K. marxianus* strains. The maximum ethanol yields of BUNL-21, DMKU3-1042 and *S. stipitis* were 0.15, 0.09 and 0.31 g/g, respectively. At 37 °C, *S. stipitis* could neither grow nor produce ethanol, but both *K. marxianus* strains grew well at this temperature. The time required to reach the maximum ethanol level was shorter, but the maximum ethanol yields were slightly lower than those at 30 °C. BUNL-21 and DMKU3-1042 showed maximum ethanol yields of 0.14 and 0.07 g/g, respectively, and they completely consumed Xyl within 48 h.

**Table 1:** Comparison of xylose consumption, ethanol formation, and xylitol and acetic acid accumulation among various strains of *K. marxianus*

Strains	Inhibitor	Temp. (°C)	Xylose conc. (g/l)	Xylose consumption (g/l)	Time (h)	Dry cell weight (g/l)	Ethanol production (g/l)	Xylitol production (g/l)	Acetic acid production (g/l)	Dry cell yield (g/g)	Ethanol yield (g/g)	Xylitol yield (g/g)	Acetic acid yield (g/g)	Reference
*S.stipitis*														
CBS 5573	–	30	20	15.0 ± 0.69 ^a^	36	4.40 ± 0.26	4.12 ± 0.61	0.00 ± 0.00	3.72 ± 0.57	0.24 ± 0.01	0.31 ± 0.15	0.00 ± 0.00	0.20 ± 0.03	This study
CBS 5573	HMF	30	20	18.7 ± 0.49	48	5.32 ± 0.52	3.73 ± 0.38	0.01 ± 0.00	0.00 ± 0.00	0.28 ± 0.01	0.20 ± 0.02	0.00 ± 0.00	0.00 ± 0.00	This study
CBS 5573	Furfural	30	20	13.91 ± 2.99	72	4.06 ± 0.21	1.82 ± 1.09	0.00 ± 0.00	0.37 ± 0.16	0.30 ± 0.05	0.12 ± 0.05	0.00 ± 0.00	0.03 ± 0.02	This study
*K. marxianus*														
BUNL-21	–	30	20	18.9 ± 0.46	48	5.29 ± 0.19	2.91 ± 0.40	0.00 ± 0.00	8.15 ± 0.98	0.28 ± 0.01	0.15 ± 0.02	0.00 ± 0.00	0.43 ± 0.04	This study
BUNL-21	–	37	20	19.1 ± 0.23	36	5.67 ± 0.48	2.58 ± 0.05	1.29 ± 0.81	7.80 ± 1.05	0.30 ± 0.03	0.14 ± 0.00	0.07 ± 0.04	0.41 ± 0.05	This study
BUNL-21	HMF	30	20	19.2 ± 0.45	48	6.65 ± 0.37	2.05 ± 0.85	0.00 ± 0.00	8.19 ± 1.14	0.35 ± 0.03	0.10 ± 0.04	0.00 ± 0.00	0.43 ± 0.05	This study
BUNL-21	HMF	37	20	18.1 ± 0.27	36	5.68 ± 0.18	2.33 ± 0.02	1.57 ± 0.44	8.57 ± 0.78	0.31 ± 0.01	0.13 ± 0.00	0.09 ± 0.02	0.47 ± 0.05	This study
BUNL-21	Furfural	30	20	18.5 ± 0.49	48	6.06 ± 0.70	2.61 ± 0.10	0.52 ± 0.31	5.28 ± 0.65	0.33 ± 0.05	0.14 ± 0.01	0.03 ± 0.02	0.29 ± 0.03	This study
BUNL-21	Furfural	37	20	19.4 ± 0.35	48	6.17 ± 0.24	2.70 ± 0.13	0.35 ± 0.53	8.16 ± 0.92	0.32 ± 0.05	0.14 ± 0.00	0.02 ± 0.02	0.42 ± 0.05	This study
DMKU3-1042	–	30	20	19.2 ± 1.09	48	7.12 ± 0.87	1.71 ± 0.45	2.22 ± 0.47	7.25 ± 0.44	0.37 ± 0.02	0.09 ± 0.03	0.12 ± 0.45	0.38 ± 0.01	This study
DMKU3-1042	–	37	20	19.3 ± 0.22	36	6.53 ± 0.69	1.29 ± 0.23	3.57 ± 0.60	6.86 ± 1.38	0.34 ± 0.04	0.07 ± 0.01	0.19 ± 0.03	0.36 ± 0.06	This study
DMKU3-1042	HMF	30	20	19.3 ± 0.42	60	7.69 ± 0.18	1.84 ± 0.24	0.78 ± 0.42	9.23 ± 1.20	0.40 ± 0.05	0.10 ± 0.01	0.04 ± 0.02	0.48 ± 0.05	This study
DMKU3-1042	HMF	37	20	19.2 ± 0.32	48	7.26 ± 0.73	1.42 ± 0.43	1.50 ± 0.94	9.10 ± 1.14	0.38 ± 0.03	0.07 ± 0.02	0.08 ± 0.05	0.47 ± 0.05	This study
DMKU3-1042	Furfural	30	20	16.8 ± 0.80	48	7.04 ± 0.11	1.76 ± 0.07	1.69 ± 0.35	5.23 ± 0.75	0.42 ± 0.03	0.10 ± 0.00	0.10 ± 0.02	0.31 ± 0.03	This study
DMKU3-1042	Furfural	37	20	17.9 ± 0.69	48	5.68 ± 0.34	1.78 ± 0.14	1.63 ± 0.36	6.67 ± 1.27	0.32 ± 0.02	0.10 ± 0.01	0.10 ± 0.02	0.38 ± 0.06	This study
SUB-80-S	–	35	20	20	48	NR	5.6	NR	NR	NR	0.28	NR	NR	Margaritis and Bajpai (1982)
80-SM-16-10	–	35	20	20	48	NR	0.26	NR	NR	NR	0.11	NR	NR	Margaritis and Bajpai (1982)
IMB3	–	45	10	10	22	NR	0.80–1.20	NR	NR	NR	0.08–0.12	NR	NR	Banat et al. (1996)
IMB4	–	45	10	~10	48	2.6	1.2	NR	NR	NR	0.12	NR	NR	Banat and Marchant (1995)
IMB4	Anaerobic	40	10	5.59	48	NR	0.53	0.40	NR	NR	0.09	0.07	NR	Wilkins et al. (2008)
IMB4	Anaerobic	45	10	3.39	48	NR	0.00	0.85	NR	NR	0.00	0.25	NR	Wilkins et al. (2008)
DMKU3-1042	–	30	20	20	72	NR	~2.60	~4.50	NR	NR	0.13	~0.23	NR	Rodrussamee et al. (2011)
DMKU3-1042	–	40	20	20	72	NR	~2.20	~6.50	NR	NR	0.11	~0.33	NR	Rodrussamee et al. (2011)
DMKU3-1042	–	45	20	~16.0	48	NR	~0.96	~3.00	NR	NR	0.06	~0.19	NR	Rodrussamee et al. (2011)

*Temp* temperature, *NR* not reported

^a^±Standard deviation of values from experiments in triplicate

During xylose utilization, xylitol was accumulated in both *K. marxianus* strains but not in *S. stipitis*. Interestingly, there was little accumulation of xylitol in BUNL-21 compared to that in DMKU3-1042 at 30 °C, which seems to be consistent with a relatively small amount of ethanol in the latter, but the xylitol concentration significantly increased in both *K. marxianus* strains at 37 °C. In addition, a large amount of acetic acid accumulated in both strains compared to that in *S. stipitis,* suggesting that the relatively low level of ethanol production in *K. marxianus* strains may be due to a high level of acetic acid accumulation. Glycerol accumulation, however, was not detected in any of the strains tested (data not shown). These findings clearly indicate that the capability of conversion of Xyl to ethanol at a high temperature in *K. marxianus* is greater than that in *S. stipitis* and suggest that BUNL-21 is preferable to DMKU3-1042 for conversion at low and high temperatures.

### Stress resistance of *K. marxianus* BUNL-21 and DMKU3-1042 and *S. stipitis*

During the fermentation process, yeast cells are exposed to several stresses including osmotic stress, ethanol stress and heat stress, all of which have severe effects on cell viability and ethanol production (Gibson et al. 2007; Puligundla et al. 2011). In addition, high temperature fermentation tends to generate oxidative stress inside cells (Zhang et al. 2015). Therefore, characteristics of not only efficient ethanol producibility but also tolerance to these stresses are potentially required for candidate yeasts for the ethanol fermentation industry.

**Fig. 2:**
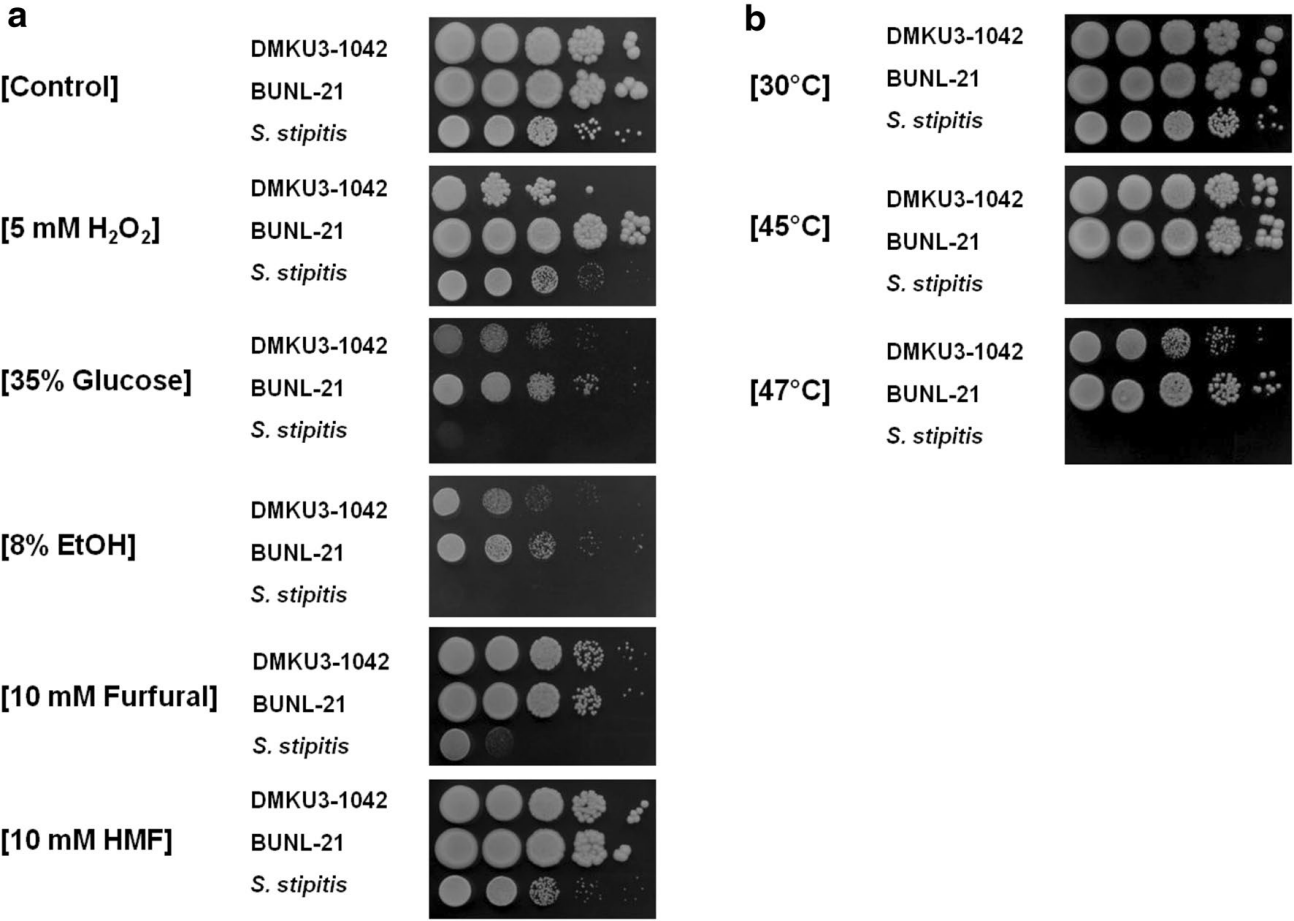
Stress resistance of *K. marxianus* BUNL-21 and DMKU3-1042 and *S. stipitis.* Analysis of stress resistance was performed as described in “Methods”. Diluted cells were spotted onto **a** YPD agar plates supplemented with 5 mM H_2_O_2_, 8 % ethanol, 35 % Glc, 10 mM furfural or 10 mM HMF. The plates were incubated at 30 °C for 48 h. **b** YPD agar medium was incubated at 30, 45 or 47 °C for 48 h

The degree of stress tolerance of BUNL-21 was thus compared with those of DMKU3-1042 and *S. stipitis*. Cells were spotted onto YPD agar plates supplemented with 5 mM H_2_O_2_ (oxidative stress), 35 % Glc (osmotic stress) or 8 % ethanol (ethanol stress), and effects of the supplements on cell growth were evaluated after incubation at 30 °C for 48 h (Fig. 2a). The three supplements were found to more strongly inhibit the growth of DMKU3-1042 and *S. stipitis* than that of BUNL-21.

The effects of furfural and HMF were also examined. The inhibitory effects of both compounds on the growth of BUNL-21 were nearly the same as those on the growth of DMKU3-1042. *S. stipitis* showed significantly weaker resistance than the two *K. marxianus* strains to furfural. BUNL-21 was also more resistant than DMKU3-1042 at 47 °C (Fig. 2b). Taken together, the results clearly show that BUNL-21 is much more robust than DMKU3-1042 under the stress conditions tested.

### Effects of furfural and HMF on growth and ethanol fermentation on Xyl

**Fig. 3:**
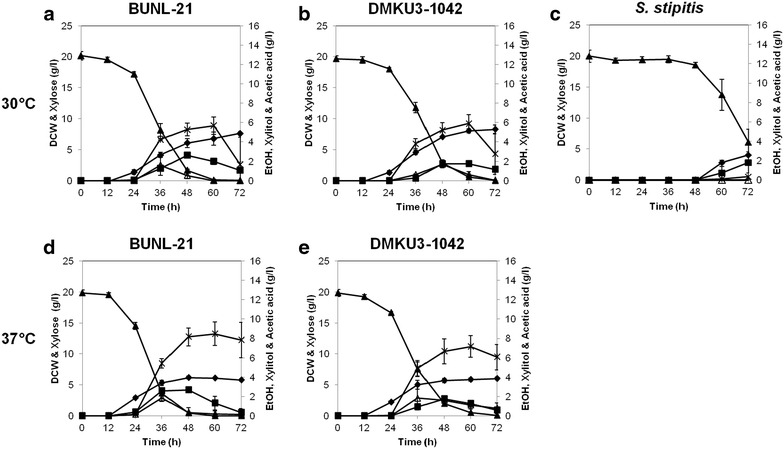
Effects of furfural on growth and ethanol fermentation on Xyl in *K. marxianus* BUNL-21 and DMKU3-1042 and *S. stipitis. K. marxianus* BUNL-21 (**a**, **d**) and DMKU3-1042 (**b**, **e**) and *S. stipitis* (**c**) were grown in YPXyl medium supplemented with 10 mM furfural at 30 °C (**a**–**c**) or 37 °C (**d**, **e**) under a shaking condition at 160 rpm, and samples were taken every 12 h until 72 h of incubation. The dry cell weight (*filled diamonds*) and concentrations of Xyl (*filled triangles*), ethanol (*filled squares*), xylitol (*open triangles*) and acetic acid (*crosses*) are indicated. *Bars* represent the ±SD of values from experiments performed in triplicate

**Fig. 4:**
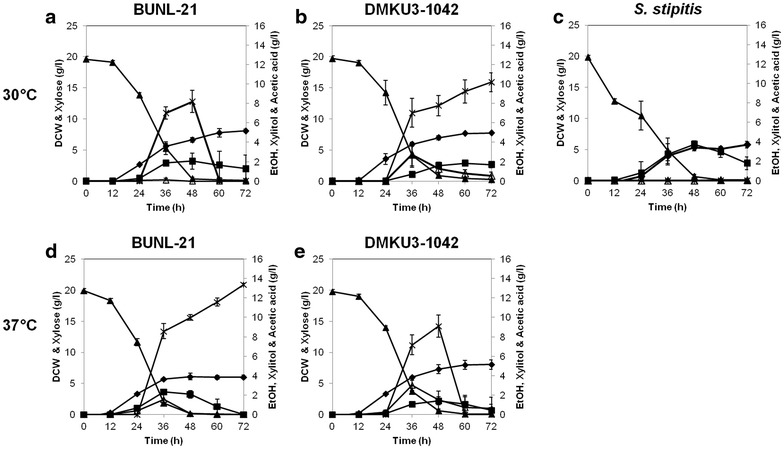
Effects of HMF on growth and ethanol fermentation on Xyl in *K. marxianus* BUNL-21 and DMKU3-1042 and *S. stipitis. K. marxianus* BUNL-21 (**a**, **d**) and DMKU3-1042 (**b**, **e**) and *S. stipitis* (**c**) were grown in YPXyl medium supplemented with 10 mM HMF at 30 °C (**a**–**c**) or 37 °C (**d**, **e**) under a shaking condition at 160 rpm, and samples were taken every 12 h until 72 h of incubation. The dry cell weight (*filled diamonds*) and concentrations of Xyl (*filled triangles*), ethanol (*filled squares*), xylitol (*open triangles*) and acetic acid (*crosses*) are shown.*Bars* represent the ±SD of values from experiments performed in triplicate

The effects of furfural and HMF on growth and ethanol fermentation on Xyl in *K. marxianus* BUNL-21, DMKU3-1042 and *S. stipitis* were further examined in liquid media at 30 and 37 °C (Figs. 3, 4, Table 1). Study of *S. stipitis* at 37 °C was omitted due to it being a non-permissive temperature for this yeast (Fig. 1). Furfural and HMF showed strong or slightly negative effects, respectively, on growth and Xyl utilization of *S. stipitis* but hardly any or very slight negative effects on BUNL-21 and DMKU3-1042. The timing of ethanol accumulation in the medium was retarded in the presence of the two compounds. At 30 °C, the maximum ethanol yields of BUNL-21, DMKU3-1042 and *S. stipitis* were 0.10, 0.10 and 0.20 g/g, respectively, in YPXyl medium supplemented with 10 mM HMF, and they were 0.14, 0.10 and 0.12 g/g, respectively, in the same medium supplemented with 10 mM furfural. At 37 °C, maximum ethanol yields of BUNL-21 and DMKU3-1042 were 0.13 and 0.07 g/g, respectively, in YPXyl medium supplemented with 10 mM HMF, and they were 0.14 and 0.10 g/g, respectively, in the same medium supplemented with 10 mM furfural. These findings and data shown in Fig. 2a suggest that both strains, especially BUNL-21, are relatively resistant to the typical toxic compounds derived from lingocellulosic biomass.

### Effect of 2-DOG on sugar utilization

**Fig. 5:**
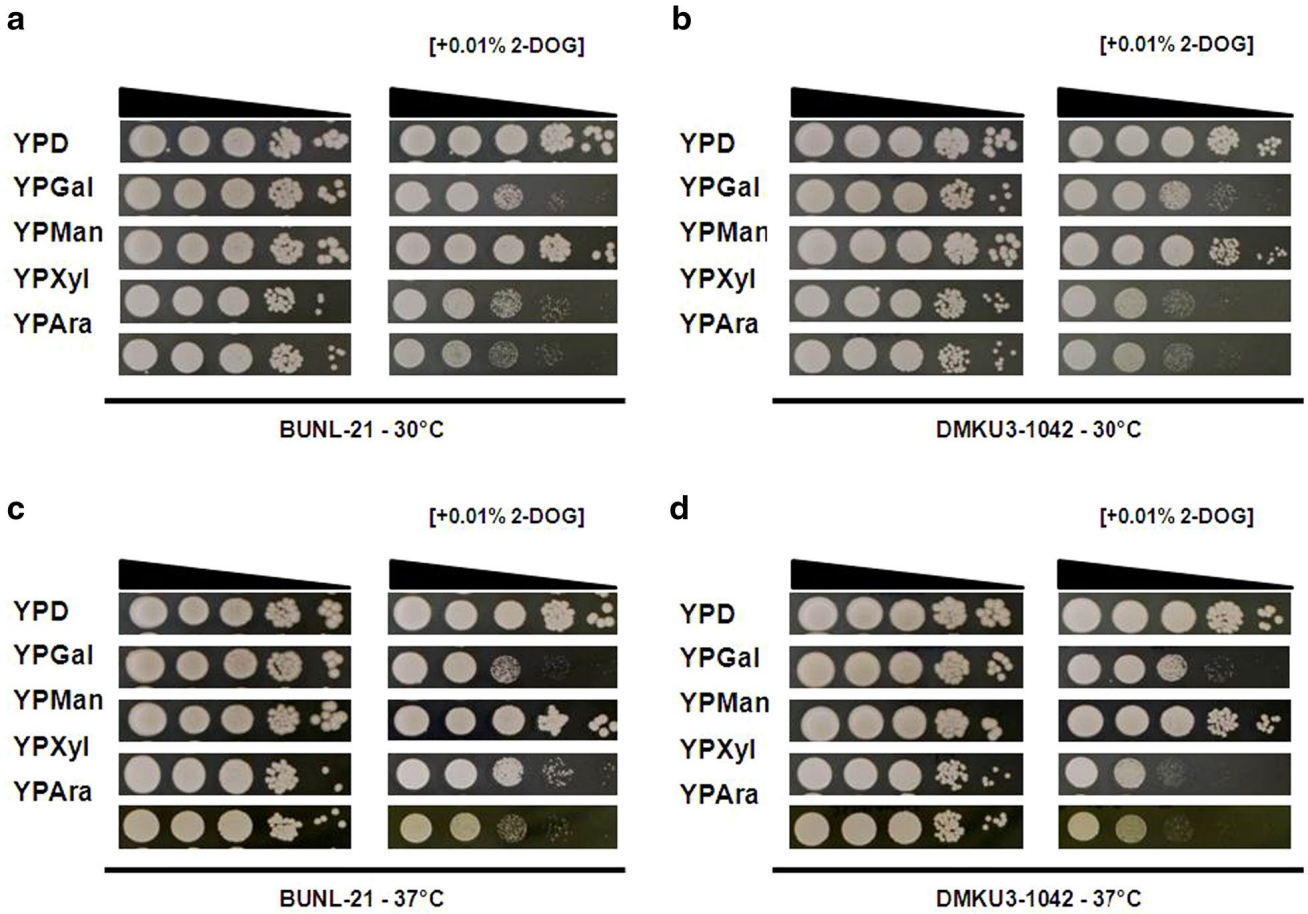
Effect of 2-DOG on utilization of other sugars at different temperatures in *K. marxianus* BUNL-21 and DMKU3-1042. Analysis of the effect of 2-DOG on utilization of other sugars was performed as described in “Methods”. Diluted cells were spotted onto YPD, YPGal, YPMan, YPXyl, and YPAra agar plates supplemented with or without 0.01 % 2-DOG. The plates were incubated at 30 °C (**a**, **b**) or 37 °C (**c**, **d**) for 48 h

Glucose repression of the utilization of other sugars is one of the disadvantages for ethanol fermentation when biomass containing several sugars in addition to Glc is used. Thus, the effect of 2-DOG as an analog of Glc on sugar utilization was examined. Cells were spotted onto agar plates of YPGal, YPMan, YPXyl and YPAra with and without 0.01 % 2-DOG and were incubated at 30 and 37 °C (Fig. 5). In the absence of 2-DOG, *K. marxianus* BUNL-21and DMKU3-1042 grew better on YPMan and YPGal than on YPXyl and YPAra at both temperatures. In the medium supplemented with 0.01 % 2-DOG, the growth on YPGal, YPXyl and YPAra, but not that on YPMan, significantly decreased. The inhibitory effect of 2-DOG on DMKU3-1042, but not that on BUNL-21, was slightly enhanced with an increase in temperature. In addition, BUNL-21 showed better growth than that of DMKU3-1042 on YPXyl and YPAra plates supplemented with 2-DOG. These results suggest that *K. marxianus* BUNL-21 exhibits a relatively weak glucose repression, especially of Xyl or Ara utilization, and is thus more suitable than DMKU3-1042 for application of lignocellulosic biomass.

### Effect of Glc on Xyl utilization

**Fig. 6:**
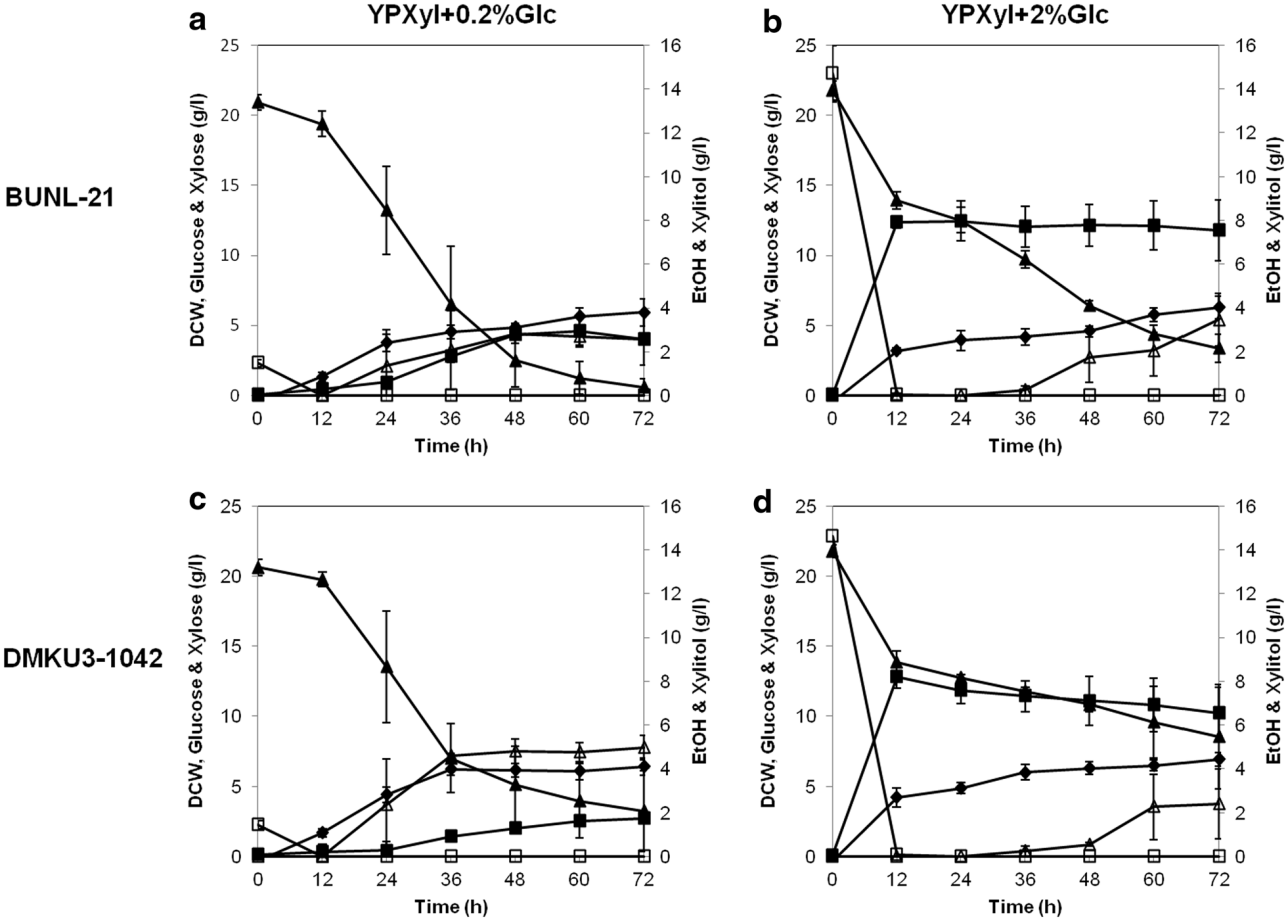
Growth and metabolite profiles of *K. marxianus* BUNL-21 and DMKU3-1042 in a mixed sugar medium. *K. marxianus* BUNL-21 (**a**, **b**) and DMKU3-1042 (**c**, **d**) were grown in 30 ml of mixed sugar YP medium of 2 % Xyl + 0.2 % Glc (**a**, **c**) or 2 % Xyl + 2 % Glc (**b**, **d**) at 30 °C under a shaking condition at 160 rpm, and samples were taken every 12 h until 72 h of incubation. The dry cell weight (*filled diamonds*) and concentrations of Xyl (*filled triangles*), Glc (*open squares*), ethanol (*filled squares*) and xylitol (*open triangles*) are shown. *Bars* represent the ±SD of values from experiments performed in triplicate

To further analyze the glucose repression of *K. marxianus* BUNL-21 and DMKU3-1042, effects of Glc on their Xyl utilization were examined (Fig. 6). Consumption patterns of Glc in the presence of Xyl were similar to those in the absence of Xyl (data not shown), and Glc in 0.2 and 2 % Glc YP media was completely used within 12 h. On the other hand, when Xyl consumption with and that without 2 % Glc in YPXyl medium were compared, the utilization of Xyl was greatly delayed, especially in DMKU3-1042, in the presence of Glc (Figs. 1, 6). Consistently, in the YP medium of 2 % Xyl + 0.2 % Glc, a relatively strong effect on Xyl consumption was observed in DMKU3-1042, but the effect was relatively weak in BUNL-21, indicating that BUNL-21 consumed Xyl faster than did DMKU3-1042 both at low and high Glc concentrations. These results seem to agree with the finding that BUNL-21 exhibited weaker glucose repression than that of DMKU3-1042 in Xyl plates as shown in Fig. 5. Moreover, the maximum ethanol concentration of BUNL-21 in the mixed sugar medium was higher than that of DMKU3-1042.

## Discussion

In this study, we compared the capability for conversion of Xyl to ethanol of the thermotolerant *K. marxianus* strain BUNL-21 from Laos with that of the efficient strain DMKU3-1042 from Thailand and *S. stipitis*. Comparison of the ethanol productivity from Xyl or/and Glc (Figs. 1, 6), degrees of tolerance to various stresses (Figs. 2, 3, 4) and degree of 2-DOG tolerance indicating susceptibility to glucose repression (Figs. 2, 5) suggests that BUNL-21 has a high potential that is superior to that of DMKU3-1042 for application in ethanol production from lignocellulosic biomass. In addition, we noticed accumulation of xylitol and large accumulation of acetic acid over ethanol in *K. marxianus* but not in *S. stipitis* when Xyl was used as a carbon source. The accumulation of acetic acid seems to result in a low level of ethanol production in *K. marxianus.*

*K. marxianus* and *S. stipitis* are Crabtree-negative yeasts. Several studies have revealed that *K. marxianus* is superior to *S. stipitis* in thermotolerance and stress resistance but inferior in terms of ethanol productivity from Xyl and oxygen dependence (Jeffries and Van Vleet 2009; Lertwattanasakul et al. 2015; Signori et al. 2014)*. S. stipitis* CBS5773 and CBS 6054 achieved ethanol yields of ~0.41 g/g under both anaerobic and microaerobic conditions (Krahulec et al. 2012). Under the conditions used in this study, *S. stipitis* CBS 5773 achieved ethanol yield of 0.31 g/g at 30 °C. On the other hand, *K. marxianus* DMKU3-1042 achieved ethanol yields of 0.13, 0.11 and 0.06 g/g at 30 °C, 40 and 45 °C, respectively, under a shaking condition, but its ethanol yield was negligible under a static condition (Rodrussamee et al. 2011). *K. marxianus* IMB4 showed ethanol yields of ~0.12 g/g at 45 °C (Banat and Marchant 1995) under an aerobic condition, but its ethanol yield decreased to 0.09 g/g at 40 °C and 0.00 g/g at 45 °C under an anaerobic condition (Banat and Marchant 1995; Wilkins et al. 2008). These results suggest that *K. marxianus* has a higher oxygen dependency than that of *S. stipitis*.

As described above, the thermotolerant characteristics of *K. marxianus* are beneficial for high-temperature fermentation or robust fermentation. The two *K. marxianus* strains tested in this study were shown to be able to ferment Xyl at 37 °C, a temperature at which *S. stipitis* cannot grow. The yield of ethanol production from Xyl of *K. marxianus* varies in different strains from 0.1 to 0.28 g/g (Margaritis and Bajpai 1982; Banat et al. 1996; Wilkins et al. 2008; Rodrussamee et al. 2011) (Table 1). However, BUNL-21 achieved ethanol yields of 0.15 and 0.14 g/g on Xyl at 30 and 37 °C, respectively, which were about 1.7- and 2.0-times higher, respectively, than those of DMKU3-1042 (Table 1). Regarding ethanol yield, although the fermentation conditions were not the same, BUNL-21 showed ethanol yields higher than those of strains 80-SM-16-10, IBM3 and IBM4, the ethanol yields of which were 0.11, 0.12 and 0.12 g/g, respectively, but lower than that of strain SUB-80-S, the ethanol yield of which was 0.28 g/g at 35 °C (Banat and Marchant 1995; Banat et al. 1996; Margaritis and Bajpai 1982). Moreover, it is notable that the productivity of both strains was hardly inhibited by HMF or furfural, and the ethanol yield of BUNL-21 was 0.14 g/g in the presence of furfural (Figs. 3, 4, Table 1).

It was found that both *K. marxianus* strains accumulated larger amounts of xylitol than that accumulated by *S. stipitis.* The accumulation of xylitol seems to reduce the yield of ethanol. We have limited evidence regarding the superiority of BUNL-21 to DMKU3-1042. BUNL-21 accumulated a smaller amount of xylitol both at 30 and 37 °C (Fig. 1). Consistently, when xylitol was used as a carbon source, BUNL-21 assimilated xylitol and grew better than DMKU3-1042 did (Additional file 1: Fig. S2). Notably, the expression level of *ADH2*, which is a gene for the major alcohol dehydrogenase in ethanol fermentation on Glc (Lertwattanasakul et al. 2007) and is down-regulated on Xyl (Lertwattanasakul et al. 2015), in BUNL-21 was found to be about 3-times higher than that in DMKU3-1042 on Xyl (Additional file 1: Fig. S3). Its relatively high expression level of *ADH2* may provide more NAD^+^ to xylitol dehydrogenase to prevent xylitol accumulation and produce more ethanol. Moreover, it was found that a higher temperature caused more accumulation of xylitol in both *K. marxianus* strains (Fig. 1). The accumulation might be due to the limitation of NAD^+^ as a result of the so-called cofactor imbalance (Jeffries and Jin 2004; Zhang et al. 2013; Hou et al. 2014). If so, it is assumed that some enzyme activity coupled to NADH oxidation is weakened at a high temperature. Alternatively, the pathway from Xyl uptake to xylitol might be enhanced or the pathway downstream from xylitol might be weakened as temperature increased.

**Fig. 7:**
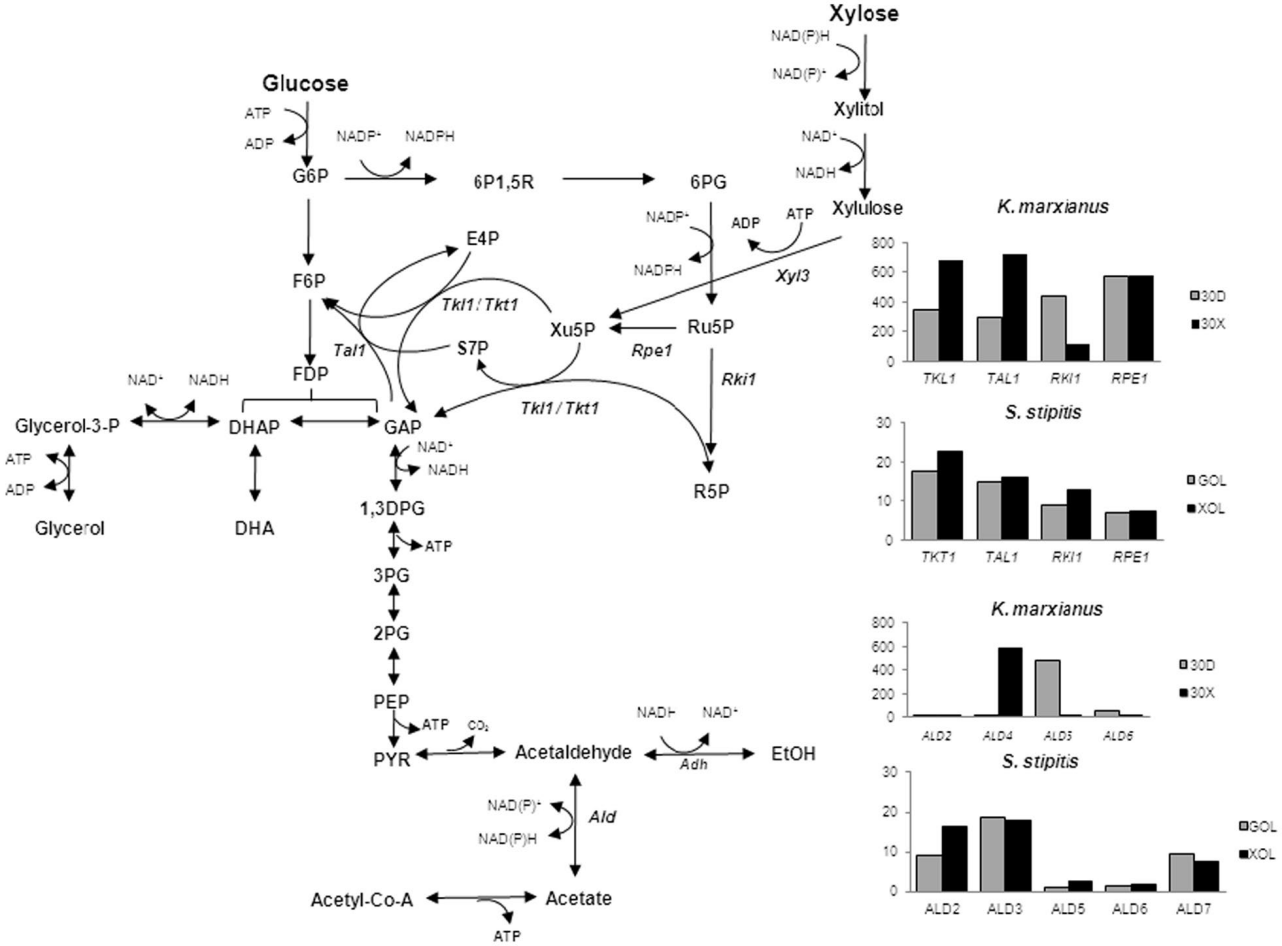
Expressional difference in genes for enzymes in the pentose phosphate pathway and acetaldehyde dehydrogenase between *K. marxianus* and *S. stipitis.* The data for *K. marxianus* and *S. stipitis* were reproduced from Lertwattanasakul et al. (2015) and Jeffries and Van Vleet (2009), respectively. Abbreviations are 30D: in Glc medium at 30 °C under a shaking condition; 30X: in Xyl medium at 30 °C under a shaking condition; GOL: in Glc medium at 30 °C under an oxygen-limited condition; XOL: in Xyl medium at 30 °C under an oxygen-limited condition

Large accumulation of acetic acid (0.36–0.43 g/g) was found in both *K. marxianus* strains at 30 and 37 °C, which may be responsible for the low level of ethanol production. One speculative reason for the acetic acid accumulation is the requirement of NADPH for xylose reductase in xylose catabolism (Signori et al. 2014). The accumulation is consistent with the up-regulation of *ALD4* for acetaldehyde dehydrogenase on Xyl (Fig. 7) (Lertwattanasakul et al. 2015). At the same time, *RKI1* for ribose-5-phosphate isomerase in the pentose phosphate pathway (PPP) is largely down-regulated (Fig. 7). We thus speculated that the NADPH supply for xylose reductase activity by PPP is limited and compensatorily the cofactor is provided by the acetaldehyde dehydrogenase-mediated reaction. On the other hand, no such down-regulation of any of the genes including *RKI1* for PPP occurs in *S. stipitis* (Jeffries and Van Vleet 2009). However, when xylulose was used as a carbon source, the level of acetic acid was hardly changed (Additional file 1: Fig. S4). Another possible reason is the supply of NADPH for removal of reactive oxygen species (ROS) via antioxidants including glutathione. Evidence that genes for the oxidative stress defense mechanism were up-regulated in Xyl medium (Lertwattanasakul et al. 2015) indicates the possibility that *K. marxianus* accumulates ROS under the condition with Xyl.

Notably, in Glc medium, large accumulation of acetic acid was observed at 45 °C, but not at 30 °C, and the accumulation of acetic acid was prevented by the addition of the reduced form of glutathione (unpublished data). These findings allow us to speculate that cells accumulate ROS at a high temperature and, via the acetic acid production pathway, supply NADPH for detoxification of ROS. In Xyl medium, however, the addition of reduced glutathione showed no effect on the accumulation of acetic acid. This failure might be due to the lower expression levels of genes for the putative glutathione transporters (*OPT1* and *OPT2*) in Xyl medium (Lertwattanasakul et al. 2015). Another possible reason for the failure is that cells require a much larger amount of NADPH for other cellular activities under the condition with Xyl as a carbon source than those with Glc at 45 °C.

## Conclusion

Application of a stress-resistant and highly efficient microbe for ethanol fermentation is a crucial point for industrial application. This study provided evidence that the newly isolated strain of *K. marxianus* BUNL-21 bears a high potential for conversion of Xyl to ethanol, has strong resistance to high temperature and to toxic materials, and exhibits relatively weak glucose repression. These beneficial characteristics will allow us to develop a more efficient Xyl-to-ethanol converter by gene engineering on the basis of BUNL-21. The first findings of a large accumulation of acetic acid and its relation to specific gene expression on Xyl in *K. marxianus* motivate us to do gene engineering to improve NADPH production and reduce acetic acid accumulation.

## Additional file

10.1186/s40064-016-1881-6 Relation of dry cell weight to OD_660_ of *K. marxianus* BUNL-21 and DMKU3 1042 and *S. stipitis*. **Fig. S2.** Growth and metabolite profiles of *K. marxianus* BUNL-21 and DMKU3-1042 in YP medium containing 2 % xylitol. **Fig. S3.** Expression of *KmADH1* and *KmADH2* genes of *K. marxianus* BUNL-21 and DMKU3-1042 in YPXyl medium. **Fig. S4.** Growth and metabolite profiles of *K. marxianus* BUNL-21 in YP medium containing 2 % xylulose.
