# Supplementary material for: Efficient conversion of xylose to ethanol by stress-tolerant Kluyveromyces marxianus BUNL-21
Source: Springerplus. 2016 Feb 27;5:185. doi: 10.1186/s40064-016-1881-6 (PMC4769242; doi:10.1186/s40064-016-1881-6)
Supplement: Supplementary file 1 — 10.1186/s40064-016-1881-6 Relation of dry cell weight to OD660 of K. marxianus BUNL-21 and DMKU3 1042 and S. stipitis. Fig. S2. Growth and metabolite profiles of K. marxianus BUNL-21 and DMKU3-1042 in YP medium containing 2 % xylitol. Fig. S3. Expression of KmADH1 and KmADH2 genes of K. marxianus BUNL-21 and DMKU3-1042 in YPXyl medium. Fig. S4. Growth and metabolite profiles of K. marxianus BUNL-21 in YP medium containing 2 % xylulose. [file 40064_2016_1881_MOESM1_ESM.pdf]

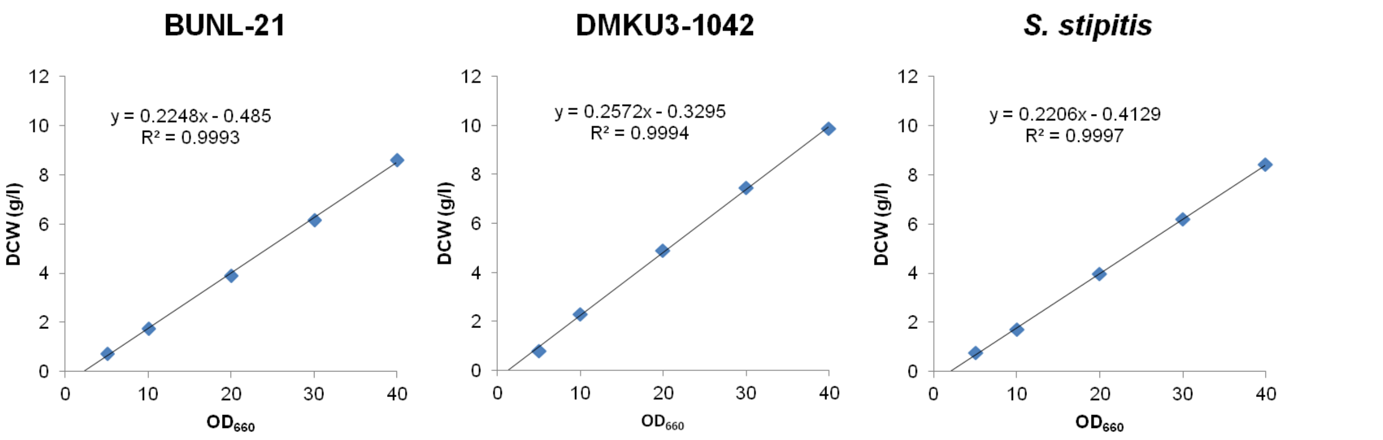

Fig. S1 Relation of dry cell weight to OD_{660} of *K. marxianus* BUNL-21 and DMKU3-1042 and *S. stipitis*

Cells were precultured in 3 ml of YPD medium. The precultures were inoculated to 100 ml fresh medium of YPD in a 200-ml Erlenmeyer flask at 30 ºC under a shaking condition at 160 rpm. The cells were harvested and washed 2 times with sterilized dH_2O. Samples were set to 1 ml of cell solution of 0, 5, 10, 20, 30 and 40 of OD_{660} in 1.5-ml test tubes, dried up at 80 ºC, and kept in a desiccator. All of the experiments were carried out in triplicate, and the values in this figure are mean values.

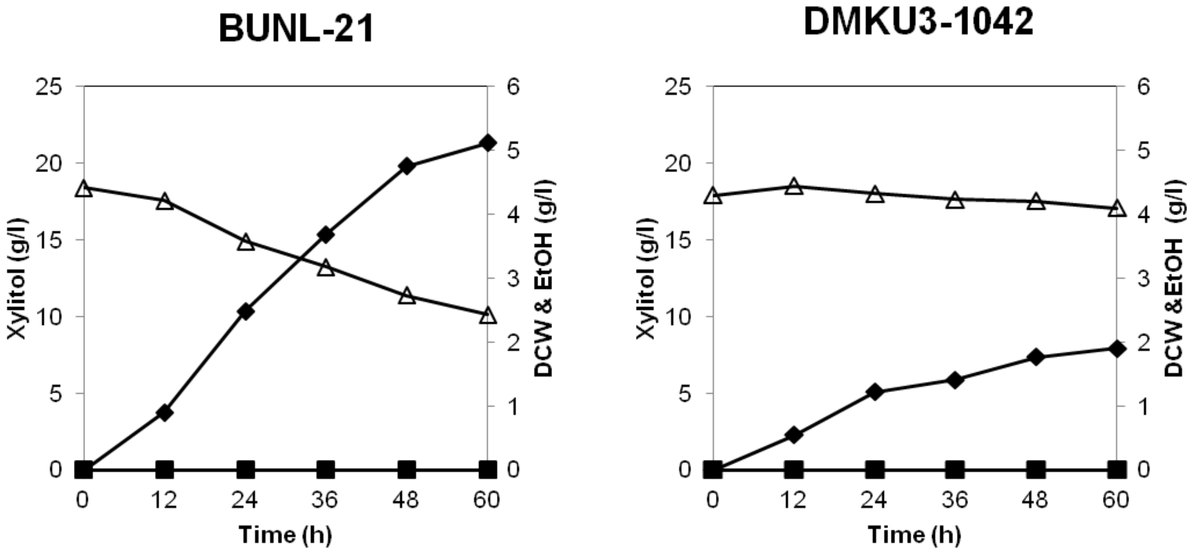

Fig. S2 Growth and metabolite profiles of *K. marxianus* BUNL-21 and DMKU3-1042 in YP medium containing 2 % xylitol

Cells were precultured in 30 ml of YPXyl medium in a 100-ml Erlenmeyer flask at 30 °C under a rotary shaking condition at 160 rpm for 18 h. The preculture was inoculated into 30-ml fresh medium of YP medium containing 2 % xylitol in a 100-ml Erlenmeyer flask, at the initial OD_{660} value of 0.1, and incubated at 30 °C under a shaking condition at 160 rpm. Samples were taken every 12 h until 72 h of incubation. The dry cell weight (*filled diamonds*) and concentrations of xylitol (*open triangles*) and ethanol (*filled squares*) are shown.

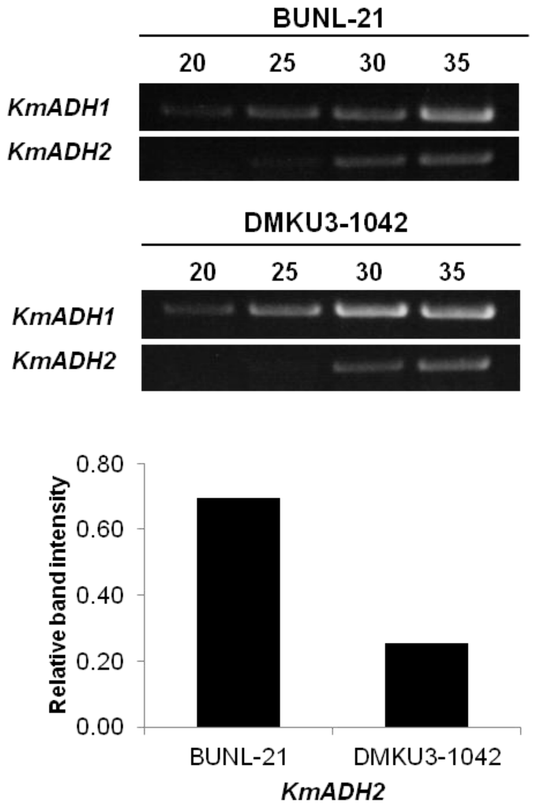

Fig. S3 Expression of *KmADH1* and *KmADH2* genes of *K. marxianus* BUNL-21 and DMKU3-1042 in YPXyl medium

Cells were precultured in 30 ml of YPXyl medium in a 100-ml Erlenmeyer flask at 30 °C under a rotary shaking condition at 100 rpm for 18 h. The preculture was inoculated to 30-ml fresh medium of YPXyl in a 100-ml Erlenmeyer flask at 30 °C under a rotary shaking condition at 100 rpm for 16 h. Total RNA was then isolated and subjected to RT-PCR. RT-PCR was performed with primers specific to *KmADH1* and *KmADH2* as previously performed (Lertwattanasakul et al. 2007). After reverse transcriptase reaction, PCR products of 20, 25, 30, and 35 cycles were subjected to agarose gel electrophoresis and stained with ethidium bromide (a). Band intensity was analyzed by using the UNSCAN-IT gel™ automated digitizing system (Silk Scientific). Ratio of intensity of each band for *KmADH2* to that for *KmADH1*, a constitutive gene, at the 30th cycle (in a liner range) is shown in (b).

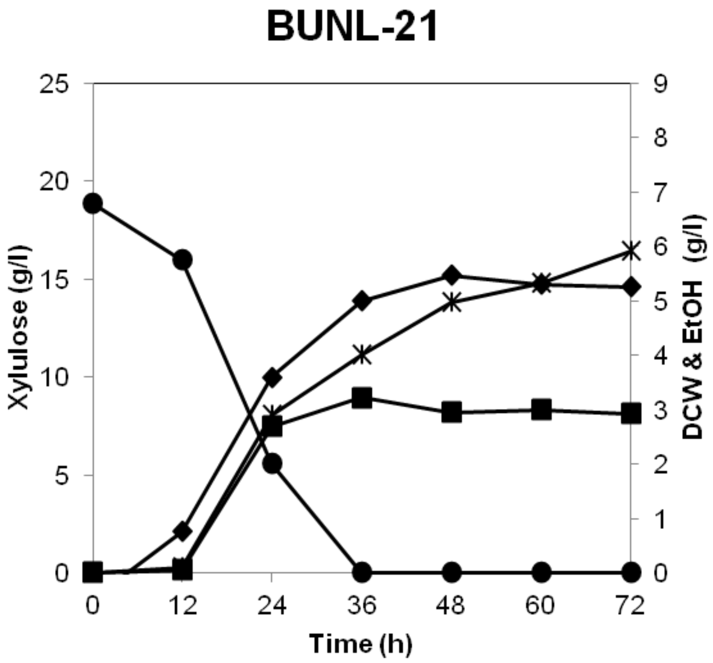

Fig. S4 Growth and metabolite profiles of *K. marxianus* BUNL-21 in YP medium containing 2 % xylulose

Cells were precultured in 30 ml of YPXyl medium in a 100-ml Erlenmeyer flask at 30 °C under a rotary shaking condition at 160 rpm for 18 h. The preculture was inoculated to 30-ml fresh medium of YP medium containing 2 % xylulose in a 100-ml Erlenmeyer flask, at the initial OD_{660} value of 0.1, and incubated at 30 °C under a shaking condition at 160 rpm. Xylulose of more than 98 % purity was prepared by purification after conversion of xylose (Tokyo Chemical Industry) by using xylose isomerase (Novozymes). Samples were taken every 12 h until 72 h of incubation. The dry cell weight (*filled diamonds*) and concentrations of xylulose (*filled circles*), ethanol (*filled squares*) and acetic acid (*crosses*) are shown.
